# Finite Element Model of the Effect of Optic Nerve Sheath Anisotropy on Ocular Loading During Horizontal Duction

**DOI:** 10.3390/bioengineering12060587

**Published:** 2025-05-29

**Authors:** Somaye Jafari, Shengqiang Cai, Joseph L. Demer

**Affiliations:** 1 Department of Ophthalmology, Stein Eye Institute, Los Angeles, CA 90095, USA; sjafari@mednet.ucla.edu; 2 Department of Mechanical and Aerospace Engineering, University of California, San Diego, CA 92093, USA; s3cai@ucsd.edu; 3 Neuroscience Interdepartmental Program, University of California, Los Angeles, CA 90095, USA; 4 Department of Neurology, University of California, Los Angeles, CA 90095, USA; 5 Department of Bioengineering, University of California, Los Angeles, CA 90095, USA

**Keywords:** anisotropic, eye movement, finite element model, optic nerve sheath, optic nerve head

## Abstract

Previous models of extraocular mechanics have often assumed isotropic properties for ocular tissues, despite evidence indicating anisotropy in the optic nerve sheath (ONS). To investigate this further, we developed a finite element model (FEM) of horizontal eye rotation using MRI data from a living subject with normal tension glaucoma. Mechanical properties were derived from tensile tests on 17 post-mortem human eyes, revealing previously unrecognized anisotropic characteristics in the ONS. We simulated ±32° horizontal eye rotations and compared isotropic versus anisotropic ONS properties using the Holzapfel model. Strain distributions in the optic nerve (ON) were analyzed using ABAQUS 2024 software. During 32° adduction, stress and strain were concentrated at the ONS-sclera junction, reaching 8 MPa and 40% with isotropic properties, and 15 MPa and 30% with anisotropic properties. In contrast, during 32° abduction, stress was lower and strain was higher in the isotropic case (6 MPa and 30%) compared to the anisotropic case (12 MPa and 25%). Increased intraocular and intracranial pressures had minimal impact on the mechanical responses. These findings suggest that the anisotropic properties of the ONS increase stress concentration at the optic disc while reducing strain during eye movements, offering new insights into ocular biomechanics. A novel phenomenon emerged from the simulations: during larger ductions, the peripapillary Bruch’s membrane is predicted to wrinkle, forming undulations with an approximately 20 μm amplitude and 100 μm wavelength at its interface with the retina and choroid.

## 1. Introduction

The optic nerve sheath (ONS) is a critical structural component of the visual apparatus, encasing the ON and playing a significant role in the mechanical load distribution during eye rotations, known as ductions. The ONS not only provides structural support but also acts as a mechanical shield, potentially protecting the ON from excessive stress and strain during ductions [1,2]. Evidence from histological studies and mechanical testing has suggested that the ONS has anisotropic properties, primarily due to the orientation of elastin fibers within it [3,4]. This anisotropy contrasts with earlier assumptions of isotropy and suggests that the ONS may have a more complex mechanical role than previously thought. Despite its importance, the mechanical behavior of the ONS remains poorly understood. This knowledge gap had broad implications because the ONS is subjected to complex loading conditions, including pressure from cerebrospinal fluid, bending, and stretching during eye movements [1,2]. Anisotropy of the ONS may influence the stress and strain distribution within the posterior eye, particularly during large-angle ductions.

Horizontal ductions, including adduction (rotation towards the midline) and abduction (rotation away from the midline), deform the posterior eye and the ON, especially for ductions exceeding 20° [5,6]. These deformations become even more pronounced in axial myopia, a form of nearsightedness in which the ocular length is increased. Previous studies have primarily focused on small-angle ductions (around 13°), which already demonstrate significant strain and deformation in the lamina cribrosa (LC) [6,7]. However, the mechanical response of the ONS during larger rotations, where the ON is more susceptible to damage, has not been thoroughly investigated. This is particularly relevant given that the structural composition of eye tissues, including the ONS, significantly influences the mechanical loads experienced during duction [3,8].

The anisotropic properties of the ONS, presumably arising from the orientation of connective tissue elements such as elastin fibers, contrast with earlier assumptions of isotropy employed in simulations of the effects of horizontal ductions [2,3]. While some experimental studies have explored the anisotropic behavior of ocular tissues such as the posterior sclera, the ONS has received less attention. For instance, Coudrillier et al. [9] used digital image correlation to analyze the stress–strain response of the posterior sclera under pressurization, revealing that the peripapillary sclera (PPS) is stiffer in the circumferential direction (around the ON) than in the meridional (longitudinal) direction. Similarly, Grytz et al. [10] employed inverse finite element modeling (FEM) to infer anisotropic material properties of the human posterior sclera and PPS based on the anatomical structure. While this approach has provided valuable insights, there are important limitations, including reliance on idealized assumptions about tissue behavior and the absence of experimental validation of the inferred properties. These limitations highlight the need for more comprehensive experimental data to accurately characterize the anisotropic behavior of ocular tissues.

To address these limitations, numerical models, particularly FEMs, have been used to simulate the mechanical behavior of ocular tissues during ductions and under varying loading conditions. These models have provided valuable insights into the stress and strain distributions within the optic nerve head (ONH) and surrounding tissues, particularly during small-angle rotations [6,11]. However, most of these FEMs have assumed isotropic properties for the ONS and other tissues, which may not accurately reflect the complex mechanical environment during large ductions. Recent FEMs have begun to incorporate anisotropic material properties, allowing for a more realistic representation of the mechanical behavior of tissues like the ONS and PPS [12,13]. The Holzapfel model, originally developed for arterial tissues, accounts for the directional dependence of mechanical properties by incorporating fiber orientation and dispersion, making it well-suited for simulating the anisotropic behavior of fiber-reinforced structures like the ONS [14,15]. These models have demonstrated that anisotropy significantly influences the mechanical response of the ONH, particularly during large ductions, where stress and strain concentrations are more pronounced.

Recent studies have provided new insight into the anisotropic behavior of the ONS. Park et al. [4] conducted preconditioned uniaxial tests on the ONS in both longitudinal and circumferential directions, revealing distinct stress–strain responses in these orientations. These findings suggest that the ONS exhibits transversely isotropic behavior, with greater stiffness in the circumferential direction compared to the longitudinal direction.

The primary aim of this study is to investigate the biomechanical impact of ONS anisotropy on ocular loading during large horizontal ductions using a finite element model (FEM). While prior models have typically assumed isotropy in the ONS, this study incorporates transversely isotropic material behavior based on newly re-analyzed experimental tensile data and implements it through the Holzapfel constitutive framework. Using anatomically accurate geometry derived from MRI of a human orbit, we simulate ±32° eye rotations to examine how anisotropic properties influence stress and strain distributions in the ONH and ONS-sclera junction, compare ON and ONS traction forces between isotropic and anisotropic models, and assess the effects of elevated intraocular pressure (IOP) and intracranial pressure (ICP). Additionally, we identify and characterize a novel mechanical phenomenon, Bruch’s membrane wrinkling, emerging during large ductions and explore its dependence on ONS material properties. Together, these objectives aim to elucidate the protective biomechanical role of ONS anisotropy and provide insights into mechanisms relevant to ON vulnerability and glaucomatous damage.

## 2. Materials and Methods

### 2.1. Anatomy

**Figure 1:**
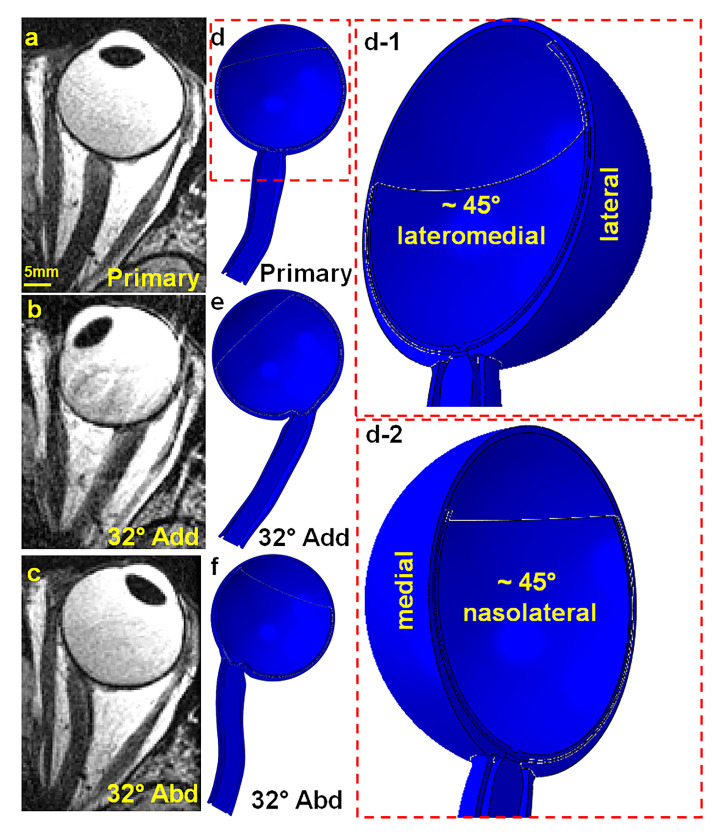
Axial MRI views of the left orbit: (**a**) primary gaze, (**b**) 32° adduction, and (**c**) 32° abduction. Hemisymmetric axial view generated by ABAQUS for (**d**) primary gaze (**d-1**) in ~45° lateromedial perspective and (**d-2**) ~45° nasolateral perspective, (**e**) 32° adduction, and (**f**) 32° abduction.

**Figure 2:**
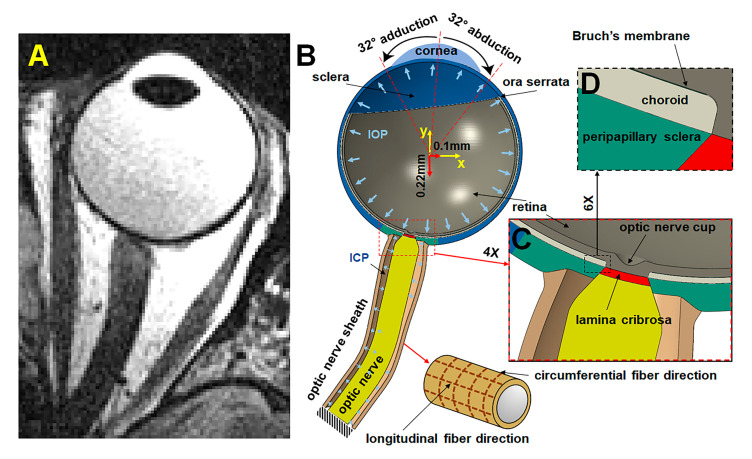
MRI and hemisymmetric eye model. (**A**) Axial MRI. (**B**) Hemisymmetric model in ABAQUS. (**C**) Magnified view of the optic nerve head region. (**D**) Further magnified view highlighting Bruch’s membrane, situated between the retina and choroid.

A realistic anatomical model of the human orbit was constructed based on surface coil magnetic resonance imaging (MRI) using a 1.5T General Electric Signa scanner. The left eye of a 72-year-old Asian female donor diagnosed with normal tension glaucoma was scanned with written informed consent under protocols approved by the local Institutional Review Board for Protection of Human Subjects and in compliance with the Declaration of Helsinki. Each MRI image had a thickness of 2 mm, with axial planes covering a 100 × 100 mm^2^ area at 390 μm resolution, and quasi-coronal planes covering an 80 × 80 mm^2^ area at 312 μm resolution, oriented perpendicular to the long axis of the orbit. Scans were performed during visual fixation of the scanned eye of a target in each of three gaze positions: central (0°, Figure 1a), large adduction (~32°, Figure 1b), and large abduction (~32°, Figure 1c). Hemisymmetric models representing primary gaze, 32° adduction, and abduction are depicted in Figure 1d,e. Figure 1(d-1,d-2) also provide the lateral–medial and nasolateral perspectives of the primary gaze model, respectively.

### 2.2. Geometry

A hemisymmetric model consisting of peripheral sclera, peripapillary sclera (PPS), choroid, Bruch’s membrane (BM), retina, LC, ON, and ONS was designed in ABAQUS 2020 (Dassault Systèmes SIMULIA Corp., Johnston, RI, USA) based on MRI of the eye (Figure 2A). Outer diameter of the sclera was set to 24 mm [16], with the equatorial sclera thinnest at ~0.42 mm and posterior sclera thickest at 0.92 mm [17,18] (Figure 2B). The diameter of PPS was set to ~8 mm based on published data [4,19]. The LC has ~1.7 mm diameter and 0.28 mm thickness. [20,21]. Thickness of the retina was set to 300 µm [22,23], and choroid was set to 200 µm [24,25,26] (Figure 2C). The thickness of the BM was taken as 5 µm (Figure 2D) [27].

### 2.3. Material Parameters for Isotropic Eye Components and Anisotropic ONS

In this study, we assume homogeneity, isotropy, and hyperelasticity for all tissues, as performed previously [7,11,13,28,29,30], except that the ONS is alternatively modeled as either isotropic or anisotropic. Although fine elastin fibers are interwoven throughout the ONS [31], ONS thickness is significantly smaller than its other dimensions [1,32,33]. Consequently, the contribution of these fibers to the stress distribution across ONS thickness is considered negligible. While some studies have characterized the retina and choroid as compressible [34,35] and the ONH as a compressible biphasic material, this study treats all tissues as incompressible, as more commonly modeled [7,29,30,36,37,38]. The isotropic tissues are described using coefficients of the reduced polynomial strain energy function *U* [4]:

(1) $$U=\sum_{n=1}^{N}C_{i0}{\left(\bar{I_{1}^{C}}-3\right)}^{n}+\sum_{n=1}^{N}\frac{1}{D_{i}}{\left(J-1\right)}^{2n},$$

where $\bar{I_{1}^{C}}$ represents the first invariant of the right Cauchy–Green deformation tensor with volume change eliminated, and J is volume ratio. $C_{i0}$ and $D_{i}$ are the coefficients of strain energy. Definitions of all parameters and variables can be found in Table 1. The number of coefficients is i, and n represents the polynomial function order. Parameters $D_{i}$s are defined in ABAQUS 2020 (Dassault Systèmes SIMULIA Corp., Johnston, RI, USA) under default settings, assuming the tissues are nearly incompressible to mitigate volumetric locking.

We previously characterized tensile properties of human ocular tissues subjected to preconditioning [4]. However, these reduced polynomial coefficients were based on a 0–15% strain range and were applied in simulations [11,13] where the incremental angle of adduction did not exceed 6° so that strain remained within 5%. In the current study, the angle of rotation ranges from 0° to 32°, and strain may exceed 30%. Therefore, the coefficients associated with the lowest degree of the polynomial were recalculated by curve fitting to the raw data [4] for a strain range of 0–50% for all tissues except the ONS. Nevertheless, all coefficients of determination (R²) describing goodness of fit to the data exceeded 0.98 (Table 2).

The previously published stress–strain curve for the ONS averaged the stress–strain curves for specimens oriented in both circumferential and longitudinal directions [4]. For the current study, the raw tensile stress–strain data from the ONS were re-analyzed to determine separate circumferential and longitudinal stress–strain curves to describe the anisotropic ONS, and the averages of the responses of specimens in both orientations were used to describe the ONS as isotropic.

**Figure 3:**
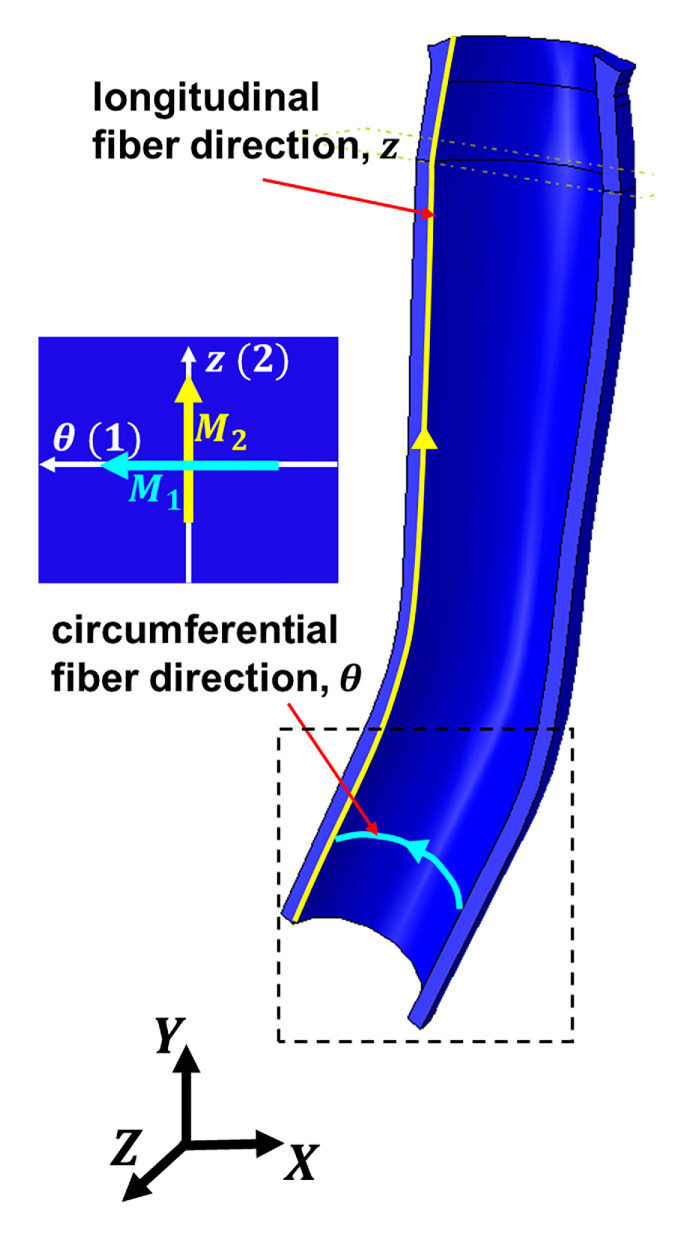
Local orientation of elastin fibers within the optic nerve sheath as defined in ABAQUS. Local longitudinal fiber direction is represented by the yellow unit vector $M_{1}$, while local circumferential fiber direction (as shown in dashed box) is indicated by the aqua unit vector $M_{2}$.

Fibers within the ONS are oriented circumferentially (Figure 3, aqua color) and longitudinally (Figure 3, yellow). In Figure 3, a local coordinate system in ABAQUS software represents fiber orientations. Unit vectors corresponding to the circumferential and longitudinal directions within the ONS are denoted as $M_{1}$ and $M_{2}$, respectively.

The constitutive formulation of Holzapfel et al. [14] was used to model an anisotropic ONS. Since the ONS is assumed to be incompressible, $\lambda_{\theta }$ and $\lambda_{z}$ in radial, circumferential, and longitudinal directions, respectively, are all assumed to be unified. Strain energy, Ψ in the Holzapfel model [23] is given by

(2) $$\Psi =\Psi_{vol}\left(J\right)+\Psi_{iso}({\bar{I}}_{1},{\bar{I}}_{4},{\bar{I}}_{6}),$$

where $\Psi_{vol}$ and $\Psi_{iso}$ are volumetric and isochoric terms of strain energy Ψ. Assuming incompressibility, $\Psi_{vol}$ represents a Lagrangian multiplier enforcing the kinematic constraint [42]. J is the elastic volume ratio. ${\bar{I}}_{1}$ is the first deviatoric strain invariant defined as ${\bar{I}}_{1}=tr\bar{C}$, where $\bar{C}$ is volume-preserving deformation:

(3) $$\bar{C}=J^{-2/3}C,$$

C is the right Cauchy–Green tensor. ${\bar{I}}_{4}=M_{1}.\bar{C}.M_{1}$ and ${\bar{I}}_{6}$ = $M_{2}.\bar{C}.M_{2}$. Under the assumption that there are no radially directed fibers, three invariants can be expressed as

(4) $${\bar{I}}_{1}={\bar{\lambda }}_{\theta }^{2}+{\bar{\lambda }}_{z}^{2}+{\left({\bar{\lambda }}_{\theta }{\bar{\lambda }}_{z}\right)}^{2},$$

where ${\bar{\lambda }}_{\theta }$ = $J^{-1/3}\lambda_{\theta }$ and ${\bar{\lambda }}_{z}$ = $J^{-1/3}\lambda_{z}$ are the isochoric stretches.

(5) $${\bar{I}}_{4}={\bar{\lambda }}_{\theta }^{2}{cos}^{2}\alpha_{1}+{\bar{\lambda }}_{z}^{2}{sin}^{2}\alpha_{1},$$

(6) $${\bar{I}}_{6}={\bar{\lambda }}_{\theta }^{2}{cos}^{2}\alpha_{2}+{\bar{\lambda }}_{z}^{2}{sin}^{2}\alpha_{2},$$

where $\alpha_{1}$ and $\alpha_{2}$ denote the angle between circumferential and longitudinal fibers, respectively, relative to local θ-axis (Figure 3). Therefore,

(7) $${\bar{I}}_{4}={\bar{\lambda }}_{\theta }^{2},\;{\bar{I}}_{6}={\bar{\lambda }}_{z}^{2},$$

The components of the Holzapfel strain energy are specified as follows:

(8) $$\Psi_{vol}=\frac{1}{D}\left[\frac{J^{2}-1}{2}-lnJ\right],$$

(9) $$\Psi_{iso}=C_{10}\left({\bar{I}}_{1}-3\right)+\frac{k_{1}}{2k_{2}}\sum_{i=1}^{2}\left\{\exp\left[k_{2}{\langle {\bar{E}}_{i}\rangle }^{2}\right]-1\right\},$$

The first term of Equation (9) represents isotropic, while the second term represents anisotropic part of the strain energy. The parameter D that is related to bulk modulus of the ONS was set to be $D=0.03\;{Mpa}^{-1}$ to ensure nearly incompressible materials in the ABAQUS Explicit environment. $C_{10}$ and $k_{1}$ > 0 are constant parameters with the dimension of MPa, and $k_{2}$ > 0 is a dimensionless parameter. $\langle {\bar{E}}_{i}\rangle$ is defined as $\langle {\bar{E}}_{i}\rangle =\frac{\left|{\bar{E}}_{i}\right|+{\bar{E}}_{i}}{2}$ incorporating the positive value of ${\bar{E}}_{i}$ [33] and

(10) $${\bar{E}}_{1}=\kappa \left({\bar{I}}_{1}-3\right)+(1-3\kappa )({\bar{I}}_{4}-1),$$

(11) $${\bar{E}}_{2}=\kappa \left({\bar{I}}_{1}-3\right)+(1-3\kappa )({\bar{I}}_{6}-1),$$

κ is the fiber dispersion parameter with range of 0≤κ≤1/3, so zero represents perfectly aligned fibers, and 1/3 represents completely dispersed fibers.

The second Piola–Kirchhoff stress tensor, S, is defined as

(12) $$S=\frac{\partial \Psi }{\partial E}=2\frac{\partial \Psi }{\partial C},$$

E, the Green–Lagrange strain tensor, is E=(C−I)/2, where the in-plane components of E are $E_{\theta }=(\lambda_{\theta }^{2}-1)/2$ and $E_{z}=(\lambda_{z}^{2}-1)/2$.

Based on Equation (12), the circumferential and longitudinal components of the second Piola–Kirchhoff stress tensor are

(13) $$S_{\theta }=\frac{1}{\lambda_{\theta }}\frac{\partial \Psi }{\partial \lambda_{\theta }},$$

and

(14) $$S_{z}=\frac{1}{\lambda_{z}}\frac{\partial \Psi }{\partial \lambda_{z}},$$

respectively.

The second Piola–Kirchhoff stress tensor is related to the Cauchy stress tensor, ${[\sigma }_{cauchy}]$, by $[\sigma_{cauchy}]=J^{-1}FSF^{T}$, while F is the deformation gradient.

Based on previously reported raw data from circumferential and longitudinal tensile engineering stress–strain graphs [4], the Cauchy stress in each direction was calculated using the relationship between Cauchy stress and engineering stress, $\sigma_{cauchy}=\sigma_{eng}\lambda$ where $\sigma_{eng}$ is the tensile engineering stress, and λ is the corresponding stretch ratio. The computed Cauchy stresses were subsequently transformed into the second Piola–Kirchhoff stresses $S_{\theta }$ and $S_{z}$.

Employing the governing Equations (13) and (14), the theoretical expressions for $S_{\theta }$ and $S_{z}$ were derived as functions of the stretch ratios and material parameters $C_{10}$, $k_{1}$, $k_{2}$, and κ. The parameter identification was performed using the simulated annealing optimization algorithm [43] implemented through the “simulannealbnd” function in Matlab 2022a. The objective was to minimize the error between the experimentally obtained second Piola–Kirchhoff stresses $S_{\theta }^{E}$ and $S_{z}^{E}$ and the corresponding model-predicted stresses $S_{\theta }^{M}$ and $S_{z}^{M}$:

(15) $$e^{2}=\sum_{i=1}^{n}\left[w_{1}{\left(S_{\theta }^{E}-S_{\theta }^{M}\right)}_{i}^{2}+w_{2}{\left(S_{z}^{E}-S_{z}^{M}\right)}_{i}^{2}\right],$$

where n represents the number of data points, and $w_{1}$ and $w_{2}$ denote weighting factors ($w_{1}=w_{1}$ = 1.0 in this study).

Nonlinear regression analysis was conducted on the experimental circumferential and longitudinal second Piola–Kirchhoff stress–stretch curves to determine the optimal values of $C_{10}$, $k_{1}$, $k_{2}$, and κ. Alternatively, $C_{10}$ was recalculated for an isotropic ONS by averging the experimental circumferential and longitudinal stress–strain curves for the ONS and removing the anisotropic term in Equation (9).

### 2.4. Boundary Conditions

As depicted in Figure 2B, the ON and ONS are constrained where they terminate at the orbital apex. Intracranial pressure (ICP) is taken as uniform within the ONS. Boundary conditions vary across six analyzed cases, as detailed in Table 3. Cases 1–4 involve horizontal eye movements with translations of +0.1 mm horizontally and −0.22 mm vertically [44], with IOP set to 15 mmHg and ICP to 10 mmHg. Cases 1 and 2 examine 32° adduction with alternatively isotropic or anisotropic ONS configurations, while Cases 3 and 4 consider 32° abduction with similar configurations.

To examine the impact of varying IOP and ICP during central gaze, the globe’s center was fixed, and the ONS was modeled as anisotropic. In Case 5, IOP ranges from 5 mmHg (low) to 40 mmHg (high), maintaining normal ICP. In Case 6, ICP varied from 3 mmHg (low) to 30 mmHg (high), with normal IOP.

### 2.5. Numerical Analysis

All simulations were performed using Abaqus/Explicit 2020 with domain-level parallelization across 8 processors, a memory allocation of 16 GB, and an average computational running time of approximately 18 h per simulation. The element type used for all eye components was C3D8R, except for BM, which was modeled using S4R shell elements. The finite element model comprised approximately 840,000 elements. Mesh convergence was assessed by calculating the maximum principal strain during 32° adduction in the anisotropic ONS case across all tissue regions. The mesh was iteratively refined until the change in strain values between successive refinements was less than 5%. Final element sizes ranged from 5 µm in the retina to 500 µm in the ONS. Tie constraints were applied at the interfaces between the retina/BM, BM/choroid, and choroid/sclera to ensure proper mechanical coupling. A mass scaling factor was introduced based on a target time increment of Δt = 1 ms to enhance computational efficiency while maintaining numerical stability and accuracy. To maintain quasi-static conditions, the kinetic energy (ALLKE) was continuously monitored and kept below 5% of the total internal energy (ALLIE) throughout the simulation. The mass added to the system via scaling did not exceed 5%. Each model simulation was run for a total time of t = 0.2 s.

## 3. Results

### 3.1. Fitting Model to Experimental Results for ONS

**Figure 4:**
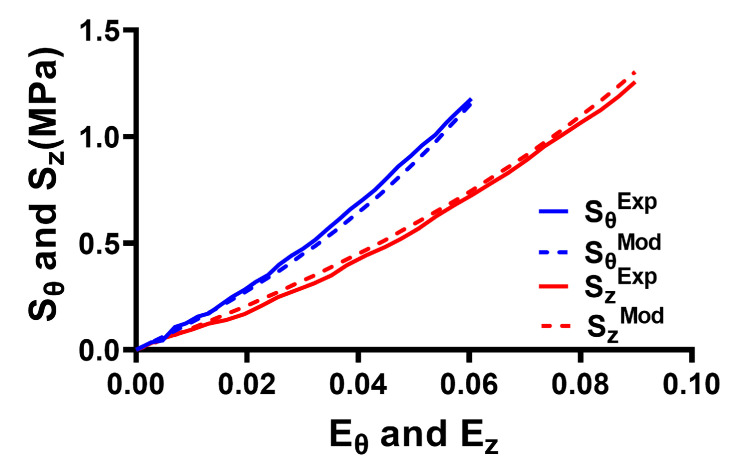
Fitting of the anisotropic Holzapfel model to experimental data for the optic nerve sheath. The X axis represents Green–Lagrange strain, and the Y axis represents the second Piola–Kirchhoff stress in the longitudinal (z) and circumferential (θ) directions. Solid lines—data. Dashed lines—model.

Figure 4 presents the results of fitting the anisotropic Holzapfel model to stress–strain data for the ONS obtained in the circumferential and longitudinal directions. Stiffness was higher in the circumferential than in the longitudinal direction. The goodness of fit of the Holzapfel model to the experimental data yielded a coefficient of determination (R^2^) of 0.99 for both the circumferential and longitudinal directions.

The resulting parameters based on curve fitting for anisotropic and isotropic ONS are summarized in Table 4.

### 3.2. On and ONS Traction Force

For an isotropic ONS, the computed ON traction forces at the ONH are 40 mN during adduction to 13°, increasing to 500 mN at 32° adduction (Table 5). Traction forces within the ONS-sclera junction are 1.5 N during adduction to 13°, increasing to 6 N at 32° adduction. During abduction, the ONH traction forces are 20 mN at 13°, increasing to 300 mN at 32°, while the traction forces within the ONS-sclera junction are 360 mN at 13°, increasing to 2.6 N at 32°.

For an anisotropic ONS, the computed ONH traction forces are 70 mN during adduction to 13°, increasing to 1.1 N at 32° adduction. The traction forces within the ONS-sclera junction are 770 mN at 13° adduction and 5.3 N at 32° adduction. During abduction, the ONH traction forces are 15 mN at 13° and 350 mN at 32°, while the traction forces within the ONS-sclera junction are 130 mN at 13°, increasing to 2.1 N at 32°.

### 3.3. Effect of ONS Anisotropy on Stress and Strain

**Figure 5:**
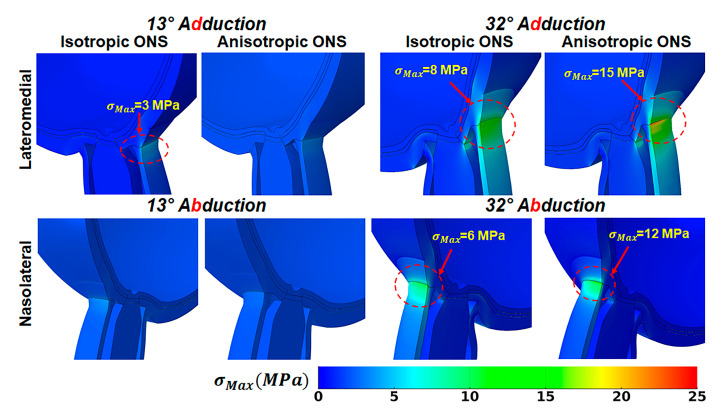
Maximum principal stress, $\sigma_{Max}$, in adduction in lateromedial perspective (**top**), and abduction in nasolateral perspective (**bottom**).

An anisotropic ONS reduced the maximum principal stress, $\sigma_{Max}$, adjacent to the ONH while increasing it within the ONS-sclera junction during horizontal ductions (Figure 5 and Table 5). In adduction, when the ONS is isotropic, $\sigma_{Max}$ in the ONH averages around 0.9 MPa at 13° and 5 MPa at 32°, while in the ONS-sclera junction, $\sigma_{Max}$ reaches 3 MPa and 8 MPa, respectively (Table 5). In contrast, with an anisotropic ONS, stress levels drop to 0.3 MPa and 3 MPa adjacent to the ONH and increase to 4 MPa and 15 MPa in the ONS-sclera junction at the same ductions, respectively. During abduction, $\sigma_{Max}$ at 13° is about 0.2 MPa in the ONH and 1.5 MPa in the ONS-sclera junction for isotropic cases and 0.1 MPa and 4 MPa for anisotropic cases. However, at 32°, stress in the ONS-sclera junction is significantly lower for the isotropic case (6 MPa) than the anisotropic case (12 MPa). Adjacent to the ONH, the corresponding stresses are 2 MPa for both isotropic and anisotropic cases. As shown in the stress–strain curves in Figure 4, the anisotropic ONS is stiffer in the circumferential direction than in the longitudinal direction. Consequently, the traction force in the ONS-sclera junction is higher for isotropic ONS than anisotropic ONS.

**Figure 6:**
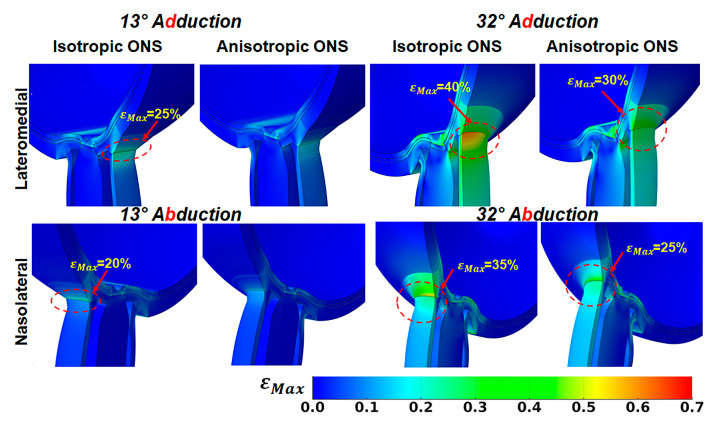
Maximum principal strain, $\varepsilon_{Max}$, in lateromedial perspective, in adduction (**top**) and abduction in nasolateral perspective (**bottom**).

Figure 6 illustrates the impact of ONS anisotropy on the distribution of maximum principal strain, $\varepsilon_{Max}$, in the ONS-sclera junction. For the isotropic ONS, average $\varepsilon_{Max}$ in the ONS-sclera junction reaches approximately 25% at 13° and 40% at 32° adduction. During abduction, these values are greatest on the medial side within the ONS-sclera junction, with strains reaching about 20% at 13° and 35% at 32° adduction.

Conversely, the anisotropic ONS results in a more uniform strain distribution within the ONS-sclera junction, resulting in lower average $\varepsilon_{Max}$ levels. During 32° adduction, strain averaged around 30% on the lateral side of the ONS-sclera junction, while during abduction, it was approximately 25% at the medial side.

### 3.4. Wrinkling During Large Angle of Duction

**Figure 7:**
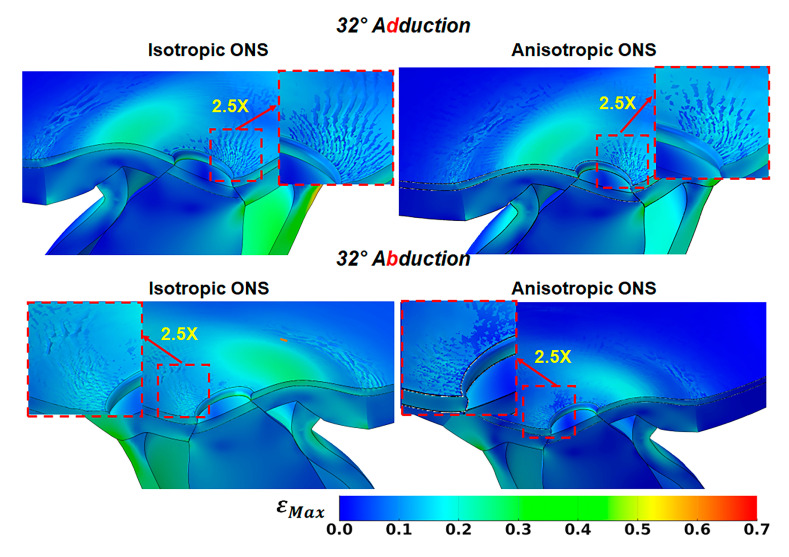
Wrinkling evidenced by variation in maximum principal strain, $\varepsilon_{Max}$, in peripapillary Bruch’s membrane at 32° adduction (**top row**) and abduction (**bottom row**). The retina has been made transparent for visualization.

From primary gaze to approximately 15° of both adduction and abduction, the interface between the retina and BM remains smooth. However, as the angle of adduction or abduction increases, the BM begins to wrinkle at the retina–BM–choroid interface in both isotropic and anisotropic ONS models. Figure 7 illustrates the emergence of BM surface wrinkling at 32° horizontal duction, with the retina rendered transparent for visualization. The average critical compressive strain initiating surface wrinkling at the optic disc during both adduction and abduction is 9% at 16° for the isotropic ONS and 5% at 13° for the anisotropic ONS. Between 13° and 32°, wrinkle wavelengths decrease as amplitudes increase.

At 32° adduction and abduction, the wrinkle wavelength is approximately 90 μm for the isotropic ONS and 100 μm for the anisotropic ONS, with amplitudes of around 6 μm and 10 μm, respectively. These differences due to isotropy are insubstantial at the optic nerve disc. However, remote from the disc, ONS anisotropy reduces wrinkling at the retina–BM–choroid interface, perhaps due to decreased stress within the retina, BM, and choroid tissues.

### 3.5. Effect of Elevated IOP and ICP

**Figure 8:**
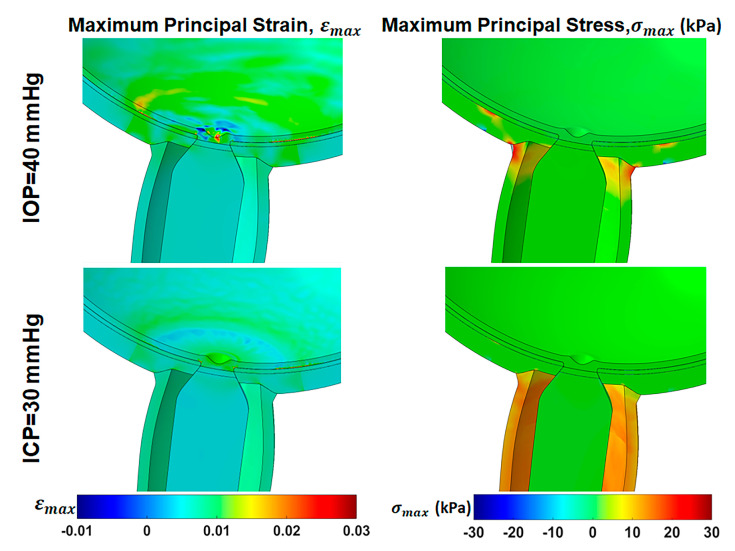
Effect of high intraocular pressure (IOP) and intracranial pressure (ICP) in primary gaze on maximum principal strain, $\varepsilon_{Max}$, and maximum principal stress, $\sigma_{Max}$, distribution in central gaze with an anisotropic optic nerve sheath.

Hydrostatic pressures were altered for comparison of their effects in primary gaze, assuming ONS anisotropy. Figure 8 demonstrates that increasing intraocular pressure (IOP) from normal of 15 mmHg (2 kPa) to 40 mmHg (5.3 kPa) results in tensile strains ranging from 0.5% within the ONS head to approximately 3% within the optic cup, retina, and peripapillary sclera (PPS), accompanied by 20 kPa (3.8 times IOP) stress within the ONH. Conversely, increasing ICP from the normal value of 10 mmHg (1.3 kPa) to 30 mmHg (4 kPa) induces tensile strains of approximately 0.5% within the ONS-sclera junction and 1.5% within the optic cup. This elevation in ICP also increases the maximum principal stress in ONS stress to approximately 20 kPa (5 times ICP).

## 4. Discussion

Reanalysis of tensile data over a wide range of strains reveals that the ONS is stiffer in the circumferential direction than in the longitudinal direction (Figure 4). This study employed a FEM based on the nonlinear Holzapfel model for the ONS to simulate horizontal ductions ranging from central gaze to as much as 32° in adduction and abduction, incorporating associated eye translations. The results highlight that anisotropy reduces traction forces within the ONS during horizontal duction, mitigating stress concentrations within the optic disc and peripapillary region.

Previous investigations primarily focused on stress and strain distributions in the posterior eye during smaller 6° to 13° rotations [6,11,13,45], rather than larger rotations where forces on the ON are much greater and may cumulatively result in ON damage [5,46]. The anisotropic ONS, being less stiff longitudinally than circumferentially, experiences less longitudinal stress than an isotropic ONS, so there is less traction on the posterior eye. Using porcine tissue properties, Wang et al. calculated ONS traction forces for 13° adduction and abduction to be 150 mN and 90 mN, respectively [6]. In the present study employing human tissue properties, anisotropic ONS traction forces were computed to be 620 mN higher in adduction and 40 mN higher in abduction at 13° than the respsective calculations by Wang et al., likely due to differences in the mechanical properties between human and porcine properties.

Earlier computational modeling incorporated anisotropic properties in the peripapillary sclera (PPS), finding that anisotropy there significantly influences ONH mechanical responses to intraocular pressure changes [47]. Experimental orientational stress–strain curves for PPS remain unavailable for application to numerical simulations. As a substitute for tensile data, some have addressed the issue using inverse finite element analysis [10] or conventional continuum frameworks [12,47] to describe tissue anisotropy. By contrast, our model integrates directly measured anisotropic properties of the human ONS, yielding more realistic predictions of stress and strain distributions during large-angle ductions and highlighting the protective biomechanical role of ONS anisotropy.

A significant prediction emerging from the current FEM is the interfacial wrinkling at the Bruch’s membrane (BM) between the retina and choroid during ductions exceeding ~15°, particularly in adduction. This wrinkling results from compression of the BM, a stiff middle layer situated between two softer layers consisting of the retina and choroid [48]. Optical coherence tomography (OCT) with a ~12 µm resolution may not detect these wrinkles predicted by the FEM to occur at 32° adduction, as the wrinkles are predicted to have an amplitude of maximum 10 µm and a wavelength of 100 µm. However, Sibony et al. demonstrated choroidal and retinal folds in papilledema whose amplitudes exceed 20 µm, with wavelengths from 107 to 530 µm [49]. The findings of our FEM, therefore, may be relevant to choroidal–retinal mechanics [50] that may be related to changes in mechanical properties [48], papilledema [49], or imbalances in scleral layer contractile forces associated with IOP [51]. Future technical improvements in OCT resolution may permit the experimental detection of the predicted wrinkles.

To compare ocular tissue mechanics under elevated IOP and ICP with their response to large horizontal eye movements, we evaluated the effects of high IOP (40 mmHg) and ICP (30 mmHg) during central gaze, assuming an anisotropic ONS. Elevated IOP or ICP results in maximum tensile strains of 0.5–3% in the ONS or optic cup and 20 kPa maximum tensile stress in the ONS junction with the sclera. Previous FEMs indicated that increasing IOP from 15 mmHg to 40–50 mmHg produces 3% average strains and 40 kPa stresses in the ONH region, depending on tissue stiffness and geometry [52,53]. Similarly, increasing ICP to 15–20 mmHg increases strain in the ONS junction with the sclera to 0.5–2% [52,54].

This study simulated quasi-static conditions that do not capture dynamic ocular responses to dynamic horizontal eye movements such as saccades. Modeling orbital fat as a viscoelastic material [55] could further clarify the behavior of the eye and ON during rapid eye movements. Nonlinear hyperelastic material properties used for ocular tissues in this study contrast with their viscoelastic nature in vivo, potentially altering stress concentrations in the ONH region following rapid rotations.

In summary, this finite element model has important clinical implications, particularly for understanding the biomechanical contributors to ONH damage in conditions like normal-tension glaucoma, where intraocular pressure is within normal limits. By simulating stress and strain distributions during large horizontal eye movements, the model can help identify mechanical risk factors in susceptible individuals, including those with axial myopia. Additionally, it may inform surgical planning in orbital and strabismus procedures by predicting the impact of anatomical changes on ONH loading. The model’s predictions, such as Bruch’s membrane wrinkling, also offer potential biomarkers for advanced imaging interpretation in clinical diagnostics.

## 5. Conclusions

The present study demonstrates that the human optic nerve sheath (ONS) exhibits significant anisotropic tensile behavior, characterized by greater stiffness in the circumferential direction compared to the longitudinal direction. Finite element simulations incorporating this anisotropy using the Holzapfel model revealed that material anisotropy substantially alters stress and strain distributions within the posterior eye during large horizontal ductions.

Quantitatively, during 32° adduction, the maximum principal stress at the ONS-sclera junction increased from 8 MPa in the isotropic model to 15 MPa in the anisotropic model, while strain decreased from approximately 40% to 30%. Similarly, the traction force at the optic nerve head (ONH) rose from 500 mN (isotropic) to 1.1 N (anisotropic), indicating increased mechanical loading localized to the sheath–sclera interface. In contrast, maximum stress adjacent to the ONH itself decreased from 5 MPa (isotropic) to 3 MPa (anisotropic), suggesting a stress-shielding effect of the anisotropic ONS.

Moreover, a novel prediction emerged: large-angle ductions (>15°) induced wrinkling at the Bruch’s membrane (BM) interface. For the anisotropic ONS case at 32° duction, wrinkle amplitudes reached ~10 µm with wavelengths of ~100 µm values potentially below the detection threshold of standard clinical OCT imaging.

These findings collectively support the hypothesis that ONS anisotropy plays a protective biomechanical role by redistributing mechanical loads away from the optic nerve head. They also underscore the importance of incorporating anisotropic tissue properties in biomechanical modeling to improve the physiological accuracy and predictive capability of simulations relevant to optic neuropathies such as glaucoma.

## Figures and Tables

**Table 1:** Definitions of reduced polynomial and Holzapfel strain energy.

$\bar{I_{1}^{C}}$	first invariant of right Cauchy–Green deformation tensor without volume change
J	volume ratio
$C_{i0}$, $D_{i}$	coefficients of strain energy
U	reduced polynomial strain energy
$\lambda_{r}$, $\lambda_{\theta }$, $\lambda_{z}$	principal stretches in radial, circumferential, and longitudinal directions
$\Psi_{vol}$, $\Psi_{iso}$	volumetric and isochoric terms of strain energy Ψ
C, $\bar{C}$	original and modified right Cauchy–Green tensor
${\bar{I}}_{1}$, ${\bar{I}}_{4}$, ${\bar{I}}_{6}$	invariants of modified right Cauchy–Green tensor
$M_{1}$, $M_{2}$	unit vectors of elastin fibers in reference state
κ	fiber dispersion parameter
$C_{10},k_{1},\;k_{2}$	parameters related to Holzapfel model
$S_{\theta }$, $S_{z}$	circumferential and longitudinal components of 2nd Piola–Kirchhoff stress tensor
$E_{\theta }$, $E_{z}$	circumferential and longitudinal components of Green–Lagrange strain tensor
$w_{1}$ and $w_{2}$	weighting factors

**Table 2:** Material parameters for ocular components.

Eye Component	Model			Reference
	2nd Order Reduced Polynomial	C_10_ (MPa)	C_20_ (MPa)	
optic nerve		0.23	8.18	
peripapillary sclera		0.20	11.60	
anterior sclera		2.25	102.20	
equatorial sclera		0.87	91.30	
posterior sclera		0.80	32.8	
	Neo-Hookean	C_10_ (MPa)	D_1_ (MPa)^−1^	
Bruch’s membrane		1.82	0.022	[39]
choroid		0.063	0.64	[40]
retina		0.003	15.5	
Lamina cribrosa	Ogden	µ_1_	α_1_	[41]
		0.36	10.4	

**Table 3:** Cases simulated.

Case	Isotropic ONS	Anisotropic ONS	32° Adduction	32° Abduction	Normal IOP and ICP	IOP Change	ICP Change
1	✓	_	✓	_	✓	_	_
2	_	✓	✓	_	✓	_	_
3	✓	_	_	✓	✓	_	_
4	_	✓	_	✓	✓	_	_
5	_	✓	_	_	_	✓	_
6	_	✓	_	_	_	_	✓

ONS—optic nerve sheath. IOP—intraocular pressure, normally 15 mmHg; ICP—intracranial pressure, normally 10 mmHg. Change of IOP was 5 mmHg (low) to 40 mmHg (high). Change of ICP was 5 mmHg (low) to 40 mmHg (high).

**Table 4:** Parameters for Holzapfel model for anisotropic and isotropic ONS.

Parameter	Magnitude	
	**Anisotropic**	**Isotropic**
$C_{10}$ (MPa)	0.8	2.3
$k_{1}$ (MPa)	32.2	-
$k_{2}$	79.1	-
κ	0.29	-

**Table 5:** Traction forces and maximum principal stresses in optic nerve head and optic nerve sheath–sclera junction.

Tissue	Optic Nerve Sheath Isotropy	Traction Force (mN)				Maximum Stress (MPa)			
		13° Add	13° Abd	32° Add	32° Abd	13° Add	13° Abd	32° Add	32° Abd
ONH	Isotropic	40	20	500	300	0.9	0.2	5.0	2.0
	Anisotropic	70	15	1100	350	0.3	0.1	3.0	2.0
ONS-Sclerajunction	Isotropic	1500	360	6000	2600	3.0	1.5	8.0	6.0
	Anisotropic	770	130	5300	2100	4.0	4.0	15	12

## Data Availability

Code is available at Zenodo 10.5281/zenodo.15226387. MRI images are available upon written application to J. L. Demer, with evidence of the local Institutional Review Board approval to receive anonymized images for research purposes.
